# Docs of the Roundtable: the use of Medical School Process Groups to Foster Healthy Professional Identity Formation

**DOI:** 10.1007/s40670-025-02584-5

**Published:** 2026-01-03

**Authors:** Ellen House, Carrie Kelly

**Affiliations:** 1 Department of Psychiatry and Health Behavior, Augusta University/University of Georgia Medical Partnership, 55 Carlton St, Athens, GA 30602 USA; 2 Department of Pediatrics, Augusta University/University of Georgia Medical Partnership, Athens, GA USA

**Keywords:** Professional identity formation, Resilience, Reflection, Undergraduate medical education, Socialization

## Abstract

**Purpose:**

Medical education should attend to student professional identity formation, the transformative process when a student incorporates the values and ideals of the profession of medicine into their own identity. A mentor-led, group reflection program was created to support medical students on their clerkships and encourage building of intentional professional identities, with the aim of sparking an ongoing practice of shared reflection and increased resilience.

**Materials and methods:**

Medical students participated in clerkship process groups (CPGs) during their 3rd and 4th year. Surveys were created and distributed to all students who participated. For the initial cohort, focus groups were utilized to gain further understanding.

**Results:**

Analysis of the qualitative data from surveys and focus groups revealed four key theme areas that were impactful regarding CPGs: development, connection, safety, and structure. *Development* reflects physician identity formation and professional development. *Connection* encompasses reconnecting with peers and caring mentors, decreasing isolation, and recognizing generalizability of experience. *Safety* refers to the maintenance of trust and confidentiality within the group. *Structure* represents logistics such as the size and timing of the sessions.

**Conclusions:**

Medical schools should adopt curricula to address professional identity formation (PIF) which (1) meet the goals of intentional physician identity development, (2) are logistically feasible for the institution, and (3) avoid adding to student stress. Clerkship process groups meet these goals. This study demonstrates they are also viewed as valuable by students, increase connection, spark reflective practices, and foster PIF. As institutions work to implement pedagogical approaches to address PIF, this study demonstrates a valuable and effective approach.

## Introduction

Medicine can be a fulfilling and joyful career, but there are also challenges intrinsic to the path of becoming and practicing as a physician. The socialization of medical students, where students learn the norms and values of being a physician and develop a sense of themselves and their place in the world of medicine, is shaped by the experiences students face and the role-modeling they witness [1, 2]. This socialization journey can affect both their well-being and professional identity formation (PIF), the transformative process when a student incorporates the values and ideals of the profession of medicine into their own identity [1, 3, 4].

Pedagogical approaches to foster PIF are recommended in medical education, and it is important to identify feasible methods to combat stress and prevent passive acculturation to negative aspects of healthcare [4–6]. It is essential to create opportunities in undergraduate medical education (UME) to help students recognize and manage the emotional burden of practicing medicine and avoid the development of maladaptive coping strategies, while also being mindful of the ever-increasing time demands medical students face [7, 8]. An internal locus of control, or sense of agency and self-efficacy in one’s life, has been associated with grit and perseverance in the face of adversity and improved mental health and wellbeing [9–11]. Curricular elements that support healthy PIF within medical education may address this by discussing a student’s agency, even within the innate hierarchy and socialization in medicine, thereby improving well-being.

As students begin their clinical rotations, the stresses, joys, and losses experienced through clinical practice, as well as their experiences with physician role-models, become more potent. In alignment with our institution’s commitment to supporting intentional professional identity formation, we sought to design a structured, reflective program to help students navigate the emotional and developmental challenges of the clerkship years. The resulting initiative, Clerkship Process Groups (CPGs), provides a faculty-facilitated space for students to share and reflect on clerkship experiences with peers and trusted mentors. The goals of the program are to normalize common challenges, reduce isolation, increase recognition of influences on development, promote healthy coping strategies, and strengthen students’ internal locus of control. Through guided reflection and discussion, CPGs aim to foster self-awareness, improve connection, and promote the lifelong reflective habits essential to resilience.

The basic structure of a CPG was formed in recognizing the value of group discussions in formative development [12–14], as well as the benefit of the presence of experienced mentors who could facilitate as well as describe their own history and growth [15, 16]. The framework for the CPGs was built on prior faculty experience with mentoring and supporting medical students’ identity formation during clerkships. To inform the creation of the CPGs, faculty preceptors and advisors detailed experiences of interacting with students and listening to challenges and questions that students experienced in their clinical years.

The theoretical framework used to develop the CPG integrated several conceptual foundations from Communities of Practice [17] and Schon’s Reflective Practice [18].

## Program Development

**Fig. 1:**
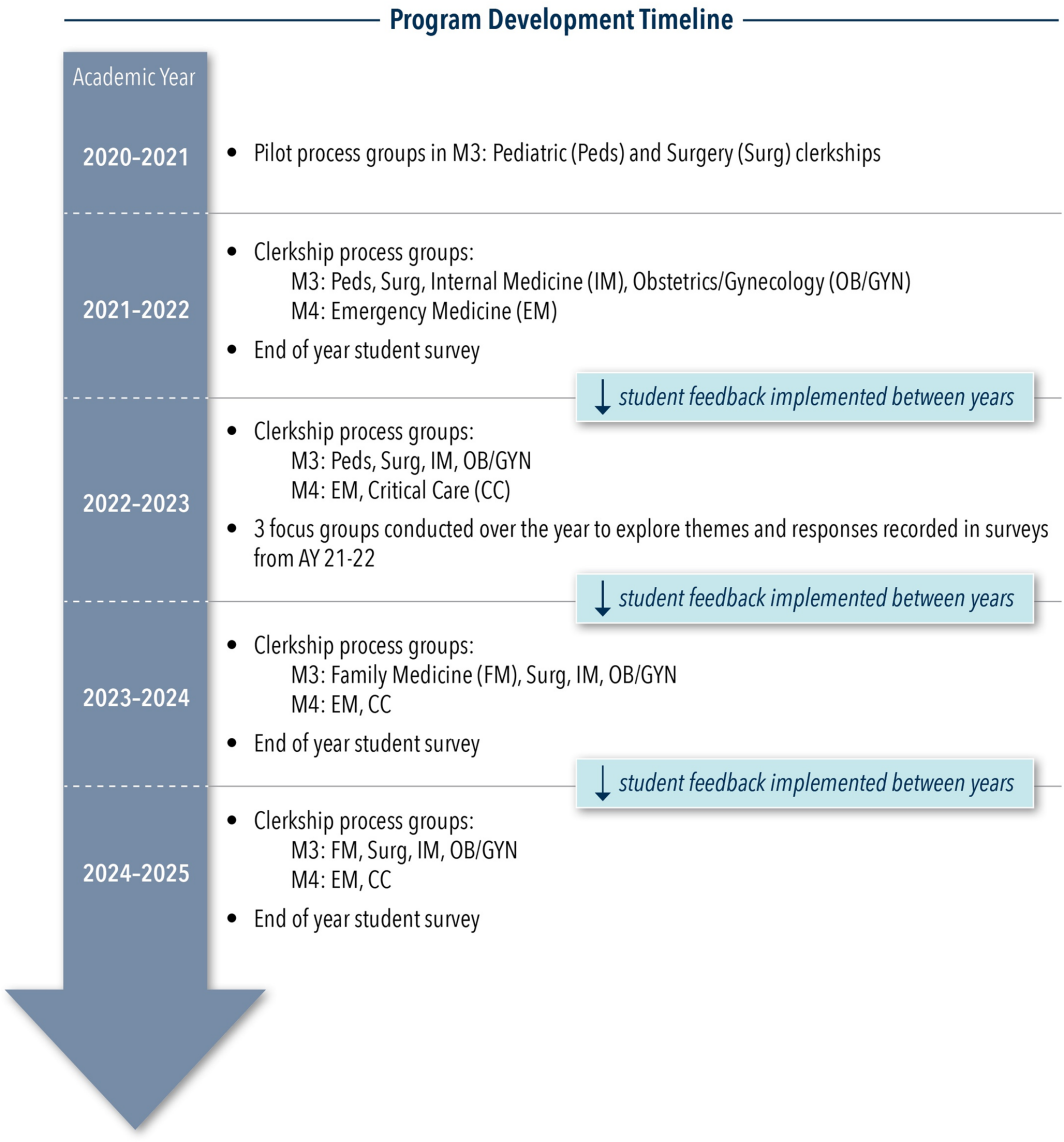
Program Development Timeline

See Fig. 1 for an overview of the timeline of the CPG program development, implementation, and data collection. Changes and improvements were made annually based on student, faculty, and clerkship director (CD) feedback to ensure benefit to students and limit disruption to clinical experiences.

Initial CPG sessions comprised one-hour group meetings of all the students (*n* = 2–10) on a clerkship rotation (e.g. pediatrics) together with a faculty facilitator. CPGs were tied to the students’ already-scheduled, mandatory, educational didactic time. As CPGs launched in the midst of the pandemic, these sessions were started in a hybrid format, with some on a virtual platform and others in-person. In conjunction with the CDs, an initial schedule and timing were arranged to limit disruption and burden to the students and their academic needs. In the pilot iteration, the CD was present in one of the CPGs. Based on early student feedback, subsequent groups were led solely by one non-grading faculty member with expertise in the field.

Initially, CPGs occurred multiple times over the course of a clerkship rotation; however, based on feedback, meetings were modified to once per rotation with more clerkships included. Timing of the group was determined to be most beneficial around the midpoint of a 4–6 week rotation where there would be experiences to discuss, but before the final week when students became more focused on their upcoming exams. The rotations selected to host a process group were determined by student feedback and indicated need, which has changed over time. In the most recent years there were clerkship process groups on the following rotations: Critical Care, Emergency Medicine, Family Medicine, Internal Medicine, Obstetrics/Gynecology, and Surgery. Participation in CPGs was voluntary during the pilot phase, but as the program developed it became a mandatory clerkship component with attendance taken. Initially there were 40 students per class and, as the school has grown, there are now 60 students per year.

Facilitators were physicians selected due to a history of strong student mentorship, expertise in the field, non-grading status, and ability to model vulnerability and discuss emotional topics comfortably [16, 19]. Facilitators met regularly in the first three years of the program development to share experiences, struggles, topics that were discussed in the sessions, and methods to improve and enrich the student experience. Facilitators remain for the entire academic year (AY), building institutional knowledge of student experiences and needs. Facilitators continue to meet officially on a biannual basis and informally throughout the year to support each other.

At the start of a clerkship students are provided a welcome letter to the CPG explaining the purpose of the group and encouraging students to be on the lookout for experiences they will want to discuss. Then to start a CPG session, the facilitator explains the structure of the upcoming hour and makes clear to the students that everything discussed is confidential outside of mandated reporting concerns (e.g., sexual harassment). Students are encouraged to come prepared with a topic or challenge from their rotation to discuss with the group, and if they are unable to produce one, the facilitator has a list of prompts to start the conversation. The list of topics were initially created by faculty members and evolved to include topics and experiences suggested by students over time. Topics include scenarios that could influence PIF (e.g. role modeling), bring up strong emotions (e.g. death of patients), cause moral injury (e.g. futile intervention at the end of life), or be ethically or interpersonally complex to navigate (e.g. responding to feedback). Facilitators remind students that the sessions are to help them develop skills to address challenges, not for the faculty facilitator or peers to “fix” problems for them, with a goal to increase a student’s sense of their autonomy and control, even in challenging times. Ideally, the students strengthen their internal locus of control as well as develop habits of reflection and leaning on colleagues, thereby improving resilience.

## Materials and methods

The conceptual framework for evaluating this program centered on reflective responses following participation in the process groups, consistent with the reactions component of the Kirkpatrick model [20].

**Fig. 2:**
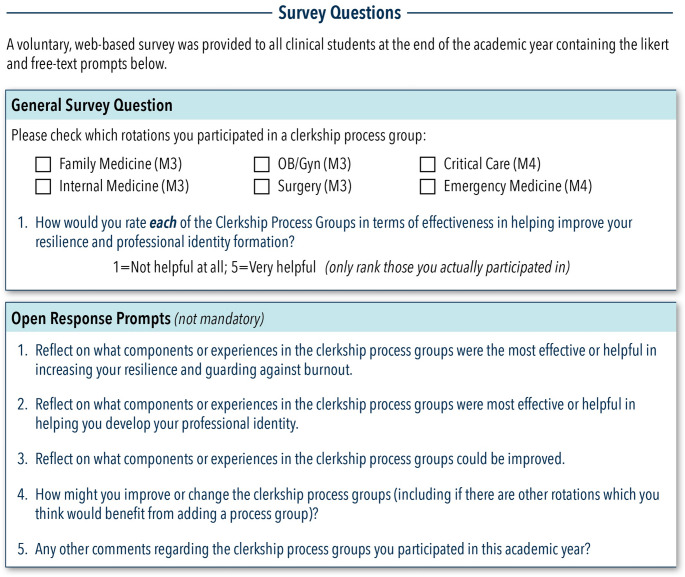
Survey Questions

Following IRB approval of the project, all students who participated in CPGs were invited to complete an anonymous, online survey with Likert scale and free response questions (Fig. 2). The first cohort (AY21-22) was also invited to participate in focus groups to explore details of survey responses. Both the survey and focus groups were voluntary, and participation in one did not imply or require participation in the other or both; $25 was provided to AY21-22 students for their participation in the survey or focus group. Focus groups (3 focus groups, total student *n* = 13) were held in AY22-23 with the first (AY21-22) cohort participants surveyed. Focus groups were prompted with the same questions as the survey with follow up on topics revealed in the initial survey if they had not yet been independently brought up by the focus group participants (i.e., student feelings on in-person versus zoom format).

A systematic approach was used to analyze the survey and focus group results from AY21-22 [21]. Both researchers inductively coded the data. They then compared codes and reconciled any discrepancies. Both of the researchers reviewed all of the codes and created categories. One researcher reviewed the categories and generated four themes to describe the overall findings. Survey data from AY23-24 and AY24-25 continue to reflect and confirm major themes identified in the surveys and focus groups from the initial cohort. The surveys had the following response rates: AY21-22 (*n* = 20); AY23-24 (*n* = 20); AY24-25 (*n* = 24).The results of these surveys have also been used in quality improvement for the CPG program.

## Results

Results from the administered surveys and the focus groups are primarily qualitative. Four key thematic areas were identified after analysis of the data: development, connection, safety, and structure. See Table 1 for specific quotes related to each thematic area; this table contains student responses from all 3 years of surveys as well as the focus groups.

**Table 1:** Themes identified from qualitative student feedback. AY21-22, AY23-24, AY24-25 surveys and AY21-22 focus groups

Theme	Description	Quotes from data that exemplify thematic feedback
Development	Impact on student professional identity formation and their role as a future physician	“It also helped facilitate my own self-reflection later on, like after the fact whether I knew it or not, I would always go back and reflect on things after the process group as well, and I don’t think I could have done that as easily if I didn’t have that prompt.” “Processing out loud with classmates in a safe setting showed me the importance of being transparent and self-aware in difficult settings, and that it’s okay to need to process things. It showed me that my professional identity IS an intersection between me as a healthcare provider and me as a human, and that that’s normal and okay.” “The simple question, “Tell me about how you handled/would handle a similar experience,” prompted me to gain ownership of my emotional and professional response to tough situations.” “Through our rotations I had already been mentally filing away things I learned from preceptors that I want to make a habit for my practice, and definitely making note of things I would have done differently. Getting this type of practice/identity honing from another 3–4 people’s experiences was super helpful.“ “I just thought that was a groundbreaking or shattering thing that I needed to hear that, even the people I, you know, are like pillars to me, also needed to learn and made mistakes.” “Sharing stories and talking about the hard stuff in medicine that sometimes a lot of people have normalized. It’s hard when a lot of these things are your first and you have no idea how to process your feelings.” “It encouraged me to think more deeply about things that I agreed or disagreed with instead of just accepting them because I am just a med student who does not know everything yet.” “While going through residency interviews, I find that having concrete examples and having said those aloud during process groups is valuable because it gives me another touch point to reflect on in preparation for interviews.”
Connection	Reconnection with peers and faculty, reduce isolation, and validate experience	“I liked hearing that other people were struggling with similar stuff I was struggling with – made things feel easier to know they were more ‘normal.’ Also appreciated hearing from faculty members their experiences and how they compared to mine.” “It’s extremely nice to have an open, non-confrontational platform to speak to our classmates about experiences we’ve had within the rotation. We see a lot of difficult things... and it’s helpful to know that we aren’t alone in the process. That our feelings aren’t weird or abnormal and that in fact, so many of our classmates feel the same way.” “Clerkships can be very isolating sometimes. Hearing shared or unique experiences of other students on the same clerkship was helpful to relieve some of the feelings of isolation. It helped with camaraderie and connection to my classmates.” “As soon as I heard my classmates start to share how they were feeling it felt like a release of like ‘Okay, you can admit that, like, everything is not okay and that you are struggling. You feel like you’re treading water and it’s okay. And at the end of the day I mean, we are all fine, but being able to like, be in that moment of panic and share it with others, not just bottle it up. By the last process groups, I was really looking forward to them.”
Safety	Maintenance of trust and confidentiality within the group	“You’re not worried that anything you say is going to affect your grade in any way, you can pretty much speak your mind.” “What made it a safe space was when the facilitator came out point blank and said (like) this is a safe space.” “It was very helpful to hear from peers about their experiences on various rotations - it made me feel less isolated in the emotional roller coaster of clerkship years. It was particularly beneficial for these experiences to take place with a moderator from faculty - it added a level of psychological protection, being able to debrief with a familiar mentor.” “A healthy feeling to it (CPG), and then I appreciate that it was, you know, a place where I could kind of just say what are good and bad, and in between.” “If I had faculty members that I knew were assessing me, I would not feel entirely comfortable that maybe my objective assessments would be safe.”
Structure	Logistics such as the size, makeup, location, frequency and timing of CPGs as well as moderation	“I liked the idea that it was an open forum and was usually led by clinicians with relevant experience in the field.” “The timing of the process group is important, halfway through or so.” “The sessions were fairly different depending on the moderator, students participating, and the questions asked. I thought that the ones with open ended questions and less structured sessions were more conducive to a productive conversation (Ex. did you have an event during your clerkship that you responded emotionally to?).” “I think if the moderators can keep track of the questions they ask and assess which ones they had best response to, that would be helpful for future sessions and how to lead them.” “I would think them being on the same rotation would be very beneficial for sure, even if it was a different group each time.” “I think Zoom is so much more convenient, but in person is so much more valuable.”

In the most recent two cohorts (AY23-24, AY24-25), students rated CPGs in terms of effectiveness in helping improve their resilience and professional identity formation. On a scale of 1–5, with 1 being “not helpful at all” and 5 being “very helpful”, the average ranking for CPGs were as follows: Family Medicine (*n* = 18) 4.28/5, Internal Medicine (*n* = 20) 4.5/5, OB/Gyn (*n* = 16) 4.44/5, and Surgery (*n* = 19) 4.21/5, Critical Care 4.33/5 (*n* = 3), Emergency Medicine 4.25/5 (*n* = 4).

### Development

The theme of *development* emerged as a central component in CPGs. This theme relates to how these sessions were impactful in student’s PIF as well as their development as a future physician, including specialty choice. Students identified CPGs as being an important prompt for reflection and professional identity development. CPGs were also identified as useful preparation for the future, including residency interviews, working together in a team, and developing a skill set to use in the future in challenging circumstances.

Other components of this theme include reflection on positive and negative role modeling that students witnessed, followed by an intentional incorporation or rejection of these behaviors. Students also noted that CPGs created space to recognize, manage, process negative feelings due to events that occur in clinical medicine instead of disconnecting from these emotions. Student responses indicate that CPGs create an active process with peers that helps PIF in a different way than a written self-reflection. CPGs give the students an opportunity to reflect on their own behavior while also listening and incorporating their colleagues’ experiences and responses.

### Connection

*Connection* was another theme that emerged as an important and valued part of CPGs. Students identified several components that CPGs provided to them that they would not have otherwise had during their clinical years. In a time of training that was identified as isolating, the student participants valued the CPGs for creating a space to maintain and create peer connections, share experiences, and validate the feelings. Students identified the groups as allowing them to celebrate wins, process mistakes and combat isolation and disconnection.

### Safety

Another major theme from the data was *safety*. Safety refers to the maintenance of confidentiality within the group, including the trust that exists between peers and the faculty facilitator. Students identified specific critical components of safety in the CPG, including a declaration of confidentiality rules which set the tone at the start of each meeting. Having faculty facilitators who were not involved in their grading or assessments was repeated as essential for creation of a sense of safety. Faculty facilitator vulnerability was also noted as contributing to a sense of safety. Students appreciated when the facilitators shared experiences and emotional responses, their own mistakes, their growth and stumbles, and affirmed experiences of the students.

### Structure

As medical students have busy schedules, the *structure* of the groups was of great importance to the participants. Logistics including the physical location of the sessions, including whether the session was virtual or in-person, and timing of the sessions were mentioned as impactful. Group dynamics including the size of the group and the group makeup were repeatedly mentioned. Variability in responses was most obvious in this theme: some students preferred larger while others smaller group size; some preferred in-person and others virtual format; some students preferred being with the same group throughout the year, while others preferred rotation specific CPGs (i.e., surgery). Overall students supported group structure that limited disruption to their clinical learning and encouraged broad participation.

Facilitation structure was also mentioned repeatedly, including how the facilitator structured the sessions and whether it was open-ended or prompts for discussion were provided. The majority of students preferred having some guidance from facilitators in the sessions, rather than having a completely open-ended conversation.

## Discussion

*“Processing out loud with classmates in a safe setting showed me the importance of being transparent and self-aware in difficult settings, and that it’s okay to need to process things. It showed me that my professional identity IS an intersection between me as a healthcare provider and me as a human, and that that’s normal and okay.”*

Medical schools recognize both the privilege and responsibility of educating the next generation of physicians, not only by imparting factual knowledge and clinical expertise, but also by preparing students to approach the rewarding and challenging realities of a career as a physician [1, 4–6]. The difference educators can make early in the identity development of medical students could reap benefits or harms for decades to come, rippling through physicians’ and patients’ lives. Findings of this study support medical schools reframing professional identity formation as an intentional, conscious process where students actively integrate personal and professional values, rather than an unconscious, passive outcome of the hidden curriculum. As faculty members hold a unique position for intervention early in student’s careers, there must also be a way to support effective, empathic, resilient development in ways that support students and do not contribute to already full loads and high anxieties [4, 16].

This innovative program builds on existing professional identity formation pedagogic methods by introducing facilitated group reflection into the clerkship curriculum; our findings indicate that group reflection provides a transformational experience different from individual or written reflective practices. CPGs harness the skills of compassionate and experienced mentors and the transformative vulnerability that group discussions can engender, supporting students and preparing them for their residencies and careers. With one facilitated hour of time on select rotations, students noted that they feel more connected, more self-aware, and more cognizant of who they want to be in their future careers. Our results show students were clear that the time in CPGs was overall valuable, which is an achievement given the multitude of pressures that students experience. Student feedback has helped identify areas in which the Clerkship Process Groups have been impactful; most notably in shaping development and professional identity formation and through increasing connection. CPGs are able to achieve these important goals because they are seen as a safe space, and careful consideration of logistics create an environment that fosters growth and reflection.

Through the process of developing the CPG sessions, a theoretical framework of Reflective Processing Communities (RPC) emerged. While using tenets of frameworks like Reflective Practice [18] and Communities of Practice [17], the RPC framework relies on the moderation of reflection and community discussion by a faculty mentor. The faculty mentor echoes the “knowledgeable other” of Vygotsky’s scaffolding practice [15] to support learners in their personal and professional development. This framework is being refined as the program evolves, but initial considerations include confidentiality, group makeup, and an open forum for discussion, with prompts if needed.

The CPG program reinforces recommendations of pedagogical approaches to address PIF in medical school [4–6], and the study also indicates that the development of a PIF program should center on the components that the students noted as significant in this research with the themes of development, connection, safety and structure. CPGs create active and conscious reflection of the events that shape the development of student’s professional identities. It is important to note that these are not “vent sessions,” but instead facilitator curated spaces for formational learning, celebration of the positive events witnessed and experienced, and increased sense of agency over who the students choose to become in their career. CPGs could foster a practice of reflection with supportive peers and mentors throughout a career.

Per the data, connection between students, and between students and faculty, helps mitigate withdrawal, isolation, and imposter syndrome that can accompany transition into clerkship years. Our data indicate that strategically placed sessions help students regularly reconnect with peers, mentors, and their own goals and values. Thoughtful choice of faculty facilitators is imperative to create an atmosphere of safety and confidentiality. Though many faculty want to be safe places for students to process their experiences, even the slightest sense that a faculty could impact a grade or possibility of matching can affect student willingness to be vulnerable. In keeping with prior research, facilitators should be individuals who students respect and who can also model vulnerability and share their own experiences of suffering, making mistakes, and effective coping strategies, which can be particularly validating and helpful for students [16, 19]. It is also important to select faculty who continue to hold joy for the practice of medicine, who approach the art of medicine with respect and appreciation rather than cynicism.

In addition to careful faculty selection, other structural components lend themselves to student engagement or distancing. Factors that deserve consideration regarding structure include when in the rotation to hold the CPG, association with other didactic time, and choice of in-person versus virtual format. Group size and makeup – for example, a longitudinal group through the year versus cohorts with each clerkship – need to be considered. Faculty facilitators should also ideally have content knowledge of the areas for which they guide discussions. Yearly feedback is also helpful to ensure that the CPG are occurring on the rotations the students deem most needed. Adjusting annually helps ensure ongoing utility to the students.

Strengths of the study include inclusion of multiple years of feedback and the use of anonymous surveys as well as focus groups. Limitations include that student experiences in any particular CPG are affected by faculty facilitation. We encourage training of new facilitators and regular meetings of facilitators to ensure ongoing benefit of the sessions. Other limitations are that we only have feedback from first year residents and current students and response rates from M4 students are consistently low. This is likely due to end of year surveys being disseminated at the same time that M4 students are graduating and transitioning to residency. We hope to address this in the future by surveying graduates on their reflections on CPG now that they are further into clinical practice. Of note, researchers are current leaders of two of the 6 clerkship process groups; however, the only component of feedback that was not anonymous was the focus groups.

In addition to surveying our graduates further into residency and clinical practice to help determine long term effects of participation in CPG, we also hope to create and publish a guide for other programs to implement and ensure ongoing process improvement. We also plan to continue to develop the theory of Reflective Processing Communities as the CPG program evolves.

The goal of sharing this work is to encourage other medical school institutions to grow their curricula addressing PIF by incorporating intentional, reflective, group practices. The results of this study show that a CPG program supports students, positively impacts professional identity formation, is viewed as valuable to students, and is readily implementable by faculty.
